# Minerals and Trace Elements Intakes and Food Consumption Patterns of Young Children Living in Rural Areas of Tibet Autonomous Region, P.R. China: A Cross-Sectional Survey

**DOI:** 10.3390/healthcare5010012

**Published:** 2017-03-01

**Authors:** Michael Dermience, Françoise Mathieu, Xiao Wei Li, Stefanie Vandevijvere, William Claus, Viviane De Maertelaer, Ghislaine Dufourny, Li Bin, Dechen Yangzom, Georges Lognay

**Affiliations:** 1 Analytical chemistry, Gembloux Agro Bio Tech—University of Liege, Passage des Deportes, 2, B-5030 Gembloux, Belgium; Georges.Lognay@ulg.ac.be; 2 Kashin-Beck Disease Fund Asbl-Vzw, Rue de l’Aunee, 6, B-6953 Forrieres, Belgium; fmathieu1@yahoo.fr (F.M.); william.claus@kbdfund.org (W.C.); 3 China National Center for Food Safety Risk Assessment, Panjiayuan Nanli, 7, Chaoyang District, Beijing 100021, China; lixw@cfsa.net.cn; 4 School of Population Health, University of Auckland, Auckland 1142, New Zealand; Stefanie.Vandevijvere@wiv-isp.be; 5 SBIM and Institut de Recherche Interdisciplinaire en Biologie Humaine et Moleculaire, Free University of Brussels, route de Lennik, 808, B-1070 Brussels, Belgium; vdemaert@ulb.ac.be; 6 CIRIHA, Haute Ecole Lucia de Brouckere, Avenue Emile Gryzon, 1, B-1070 Brussels, Belgium; gdufourny.ciriha@heldb.be; 7 Center for Disease Control and Prevention—North Lin Kuo Road 21, Lhasa 850000, China; info@kbdfund.org; 8 Kashin-Beck Disease Foundation, Gakyiling Hotel, Tuanjie Xincun, Sera Road, Lhasa 850000, China; adminkbdftib@hotmail.com

**Keywords:** 24-h food recall, cross-sectional survey, minerals, trace elements, usual intakes, preschool children, Kashin-Beck disease, Tibet Autonomous Region

## Abstract

Background and objectives: Several studies revealed clinical signs of stunting and rickets among rural populations of Tibet Autonomous Region (T.A.R.), and especially amid children. Further, these populations are affected by a bone disease named Kashin-Beck disease (KBD). However, little is known about the dietary status of this population. This survey aimed to assess the usual intakes of young Tibetan children living in rural areas around Lhasa for energy, water, and ten minerals and trace elements (Na, K, Ca, P, Mg, Fe, Zn, Cu, Mn, and Se) involved in bone metabolism. Design: A cross-sectional survey was designed. Totally, 250 pre-school children aged 3–5 years living in rural areas were enrolled. The 24-h food recall method was used to collect the intakes for two days, during two different seasons (September 2012 and April 2013). Because Tibetan foods are mainly derived from local agriculture and artisanal production, a combination of food composition tables was compiled, including specific and local food composition data. Results: The Chinese dietary recommended intakes are not met for most of the elements investigated. Intake of sodium is much too high, while usual intakes are too low for K, Ca, Zn, Cu, and Se. Bioavailability of Ca, Fe, and Zn may be of concern due to the high phytic acid content in the diet. Conclusion: These nutrient imbalances may impact growth and bone metabolism of young Tibetan children. The advantages of the implementation of food diversification programs are discussed as well as the relevance of supplements distribution.

## 1. Introduction

According to a previous study [1], populations of the Tibet Autonomous Region (T.A.R.) share four macro-ecosystems: the urban zones; the suburban zones; the agricultural zones; and the pastoral zones inhabited by nomads. Dietary habits are quite different between these groups. Nomads have a diet mainly based on dairy and meat products. Urban and suburban populations have access to a wide range of food products thanks to imports from Mainland China. Conversely, rural populations from the agricultural zones have been reported to have a very monotonous diet, based on cereal products, with a very limited variety of food products [2,3]. Several studies focusing on anthropometric measurements and nutritional status assessment have been conducted [4,5,6]. They invariably observed clinical signs of malnutrition and concluded on a poor nutritional status of the rural Tibetans, with a diet low in vegetables, meat and dairy products, but rich in cereals. More specifically, mineral deficiencies are thought to be involved in the high prevalence of rickets, and they are supposed to play a role in a little known disease called Kashin-Beck disease (KBD) [7,8]. Only one study conducted by Wang et al. (2010) [9] was found to assess the mineral intakes of rural Tibetans. They investigated calcium, iron, and zinc intakes (among other nutrients) of rural Tibetan mothers with help of food frequency questionnaires and China food composition table (FCT). Although they did not confront their results to the Chinese Dietary Reference Intakes (DRIs), they concluded that the intakes were close to sufficient intakes compared with the *2002 Chinese National Nutrition and Health Survey*. Several elements are important for healthy growth and are intimately related to bone metabolism [10]. Some major elements are required in substantial amounts, while other trace elements are needed in very small quantities. 

Considering the lack of data in the scientific literature, the main objective of this study was to assess the daily intakes for ten minerals and trace elements of dietary relevance—sodium (Na), potassium (K), calcium (Ca), phosphorus (P), magnesium (Mg), iron (Fe), zinc (Zn), copper (Cu), manganese (Mn), and selenium (Se)—among preschool children living in rural areas of T.A.R., and to compare them against the Chinese Dietary Recommended Intakes (DRIs) [11] and the harmonized Estimated Average Requirements (hEARs) [12]. The hEARs are derived from the recommended values of the most influent organizations (World Health Organization, Institute of Medicine of the U.S. National Academies of Sciences, etc.). To our knowledge, it is the first time that mineral and trace element intakes have been assessed by the 24-h food recall method in young Tibetan children living in rural areas. Because a previous study highlighted significant discrepancies between minerals concentration in the China FCT and in local Tibetan foods [11,13], and considering that the main traditional Tibetan foods are missing, a specific food composition table was compiled. It is the results of local foods sampling and analysis [14] enriched with data from the China FCT and from the USDA Food search for Windows [15]. The results of this study will provide an overview of the dietary mineral status of the rural Tibetan children and could help guiding the ongoing strategies set up by the government and by non-governmental organizations to improve the nutritional quality of the rural Tibetan diet. It is also susceptible to give some leads to further investigate the etiology of KBD.

## 2. Material and Methods

### 2.1. Ethical Standards Disclosure and Participants 

This study was conducted according to the guidelines laid down in the Declaration of Helsinki and all procedures involving human subjects/patients were approved by the Chinese Center for Disease Control and Prevention (CDC) of Tibet Autonomous Region. Verbal informed consent was obtained from the parents of the children. 

For this study, 250 preschool children aged 3–5 years old from 8 communities—Nimashangri, Nyanang, Thanggo, Theumba, Paco, Nyemo, Tharong, and Shume—located in 3 counties—Lhünzhub, Maizhokungar, and Nyêmo—of the Lhasa prefecture were randomly selected among the civil registries owned by the community leaders, proportionally to the size of the targeted population of each county. 

### 2.2. Design of the Study

As it is quantitative, and implementation is achievable considering field constraints, the 24-h food recall method has been chosen for this study. Two climatic seasons can be identified in this part of the Tibetan plateau [1], and two food consumption patterns intimately related to the seasonal variations can be observed. While fruits and vegetables are more available during summertime (May–October), an increased consumption of meat and dairy products is characteristic of wintertime (November–April). Given this information, two rounds of investigation have been conducted, the first one in September 2012, and the second one in April 2013. Except for twelve children, 238 children were interviewed twice, each time for one 24-h food recall for both seasons. All days of the week were equally represented in the final sample. 

The caretakers of the children were interviewed by six interviewers. The interviewers were local people, with a high educational level, fluent in local language and in English. They underwent an intensive two-week training inspired by the training program of Gibson and Ferguson (2008) [16]. During the campaigns, they followed a three-day workflow pattern. The first day, the interviewers met the selected children and their caretaker. They explained the study and gave them a picture chart depicting the foods most often eaten in rural Tibet. The second day, the caretakers were asked to mark the chart to indicate the foods consumed by the children in order to facilitate the recall on the next day. The third day, the caretakers and the children were interviewed together. Children were also weighed and measured. In a purpose to recall children’s exact food intake during the preceding day, the interviewers implemented a four-stage, multiple-pass interviewing method described by Gibson and Ferguson (2008) [16]. An indirect approach has been recommended, and leading questions and judgmental comments were avoided. Quantities of foods consumed were estimated principally by measuring household utensils (with water and graduated cylinder), by direct weighing, by modeling play dough molded into the correct size and shape of the food, or by using measured standard weight of food items. The interview protocol was standardized and was tested prior to the study. Adherence to the interview protocol and accuracy of food coding was checked by the field supervisor. 

### 2.3. Food Composition Data

The choice of a specific FCT is critical to achieve a good quality of results in nutritional assessment, especially when dealing with mineral and trace elements [16]. The China Food Composition Table (2nd edition, 2009) [11] appears to be the most appropriate for this study as it is the national FCTs. Moreover, it scored a quality index of 2 out of 3, according to the ratings for quality control criteria of Gibson and Ferguson (2008), reflecting a good reliability of food composition values. However, for the aforementioned reasons, the China FCT was not exhaustive enough and was completed by food composition data from Dermience et al. (2014) [14], from the United State Department of Agriculture USDA Food search for Windows [15], and from product labels. The relative percentages of food items from the different tables were 56%, 29.3%, 13.3%, and 1.4%, respectively. It is worth emphasizing that the food contributing the most to mineral intakes are coming from Dermience et al. (2014) [14], and are resulting from food sampling and analysis in the enrolled families.

### 2.4. Converting Portion Sizes to Weight Equivalents

Several procedures were used in order to convert the portion sizes into weight equivalents: (i) direct weighing of actual food or replicas by interviewers, using dietary scales; (ii) recording the volume of water that is equivalent to the volume of food and beverage; and (iii) recording household measures volumes. The recorded volumes were converted into weight equivalents by: (i) using published specific gravity data (if available); and (ii) compiling specific gravity data. For this purpose, five to eight samples of the desired food product were purchased and weighed. Then the volumes were determined by water displacement, and specific gravities (g/mL) were calculated.

### 2.5. Calculation of Food Consumption Pattern and Dietary Intake

The relative consumption of a food item were calculated based on the number of children who consumed the given food item, divided by the total number of children enrolled in the county, expressed as percentage.

Online software called *Planning Alimentaire Nubel PRO* was used to compute mineral and trace element intakes. Two days of daily intakes per child were computed for the following nutrients: energy (kJ/kCal), water (g), sodium (mg), potassium (mg), calcium (mg), phosphorus (mg), magnesium (mg), iron (mg), copper (mg), zinc (mg), manganese (mg), and selenium (µg). 

### 2.6. Statistical Analysis

All statistics were calculated using the SPSS software (v. 22.0, IBM Corp., Armonk, NY, USA) and Minitab 15 for Windows (Minitab Inc., State College, PA, USA). A *p*-value lower than 0.05 was considered for assessing significant statistical effects. In accordance with the central limit theorem and considering the important sample size, assessing the normality of the distributions was not mandatory. The Levene test was applied to detect possible non-homogeneity of the variances. Mean daily intakes between counties (3 levels) and seasons (2 levels) were compared by analysis of variance (ANOVA with two fixed factors), in order to have a general overview of the significant differences and to assess the interactions. Subsequent one fixed factor ANOVA analyses were performed for each season to compare the mean daily intakes between the counties. In the case of statistical significance, multiple comparisons tests were applied to identify means which differed from the others. In the case of homoscedasticity, the Tukey test was used. In the case of non-homogeneity of the variances, the Dunnett’s T3 test was used. Usual intakes for the group have been computed with the IMAPP software [12] and have been compared to the Estimated Average Requirements (EAR) and Upper intake levels (UL) to assess the risks of inadequate intakes.

## 3. Results and Discussion

### 3.1. Food Consumption Pattern

Table 1 shows the relative consumptions, in percentages, of different foodstuff according to the three counties and the two seasons investigated. These data confirm the still very traditional character of the rural Tibetan diet, but it also gives a glimpse of some secondary influence of the significant growth of the infrastructures in T.A.R. The main beverage is the famous yak butter tea, which is made of yak butter, tea juice or concentrated brewed black tea [14], and salt, all mixed together with boiled water. It appears that margarine is mixed with yak butter to prepare the butter tea in 4% to 56% of the families depending on the county (Table 1). The reason is that margarine is quite cheap even for rural people, while yak butter is much more valuable and is often sold in cities to generate income. Apart from yak butter tea, black tea and sweet tea are frequently consumed. Black tea is made of black tea leaves brewed with hot water and salt. Sweet tea is made of milk powder, sugar, tea leaves, and hot water. Besides, a significant percentage of the children, up to 30%–50% in Nyêmo County, consumed a homemade barley beer called *chang*. Parents are probably not aware of the harmful effects of alcohol on the children’s brain [17,18], and *chang* is given to the children to warm them up and maybe to calm them when excited. To the best of our knowledge, the consumption of soda drink by rural people of T.A.R. was never reported before in the literature. Here we can notice that sodas are part of the diet for a considerable number of children. This new habit of consumption may be correlated to the recent development of infrastructures in T.A.R., which results in easier access to various consumer goods [19].

*Tsampa* is the Tibetan name of the flour obtained from roasted barley grains. It is the main staple with 66% to 85% of the children consuming it. If it is frequently eaten under the form of *tsamtuk*, a kind of soup sprinkled with *tsampa*, it is above all consumed daily under the form of *pag*. In this traditional recipe, *tsampa* is mixed with yak butter tea and eaten as such, without cooking. Sugar, butter or dried cheese depending on availability and tastes may also be added. Although not locally grown, rice is well established in the food habits with remarkable consistency throughout the counties and the seasons. Wheat flour is also frequently consumed, mostly under the form of a traditional Tibetan noodle soup called *thukpa*, or under the form of *momo*, another traditional Tibetan dish. The latter can be plain or stuffed, steamed or fried, and depending of the amount of water incorporated into the dough, it may be compared to a bun or to a kind of ravioli. Potatoes are widely consumed by the rural Tibetan children, who eat them either as a snack between meals, or as vegetable in a dish.

The consumption of meat is generally low across all counties. Yak meat is the most consumed. However, according to local people, purebred yak have become rare in the region. Most of the livestock is the result of hybridization between a bull and a female yak, which is called *dzo* [1]. A remarkably constant consumption of pork meat throughout seasons and counties is noticeable. Contrary to yaks, goats, and mutton, raising pigs is not common in these rural areas. The consumption of pork is might also be a consequence of the infrastructural development, though pigs are also historically brought from Russia [20]. The consumption of chicken meat is very low because of the cultural reasons discussed below.

Yak or cow butter represents the main source of lipids. It is principally consumed in the butter tea, and it is more and more frequently mixed with margarine. Yak and cow fats are collected and dried when animals slaughtered, and it is used for flavoring some traditional dishes (stuffed *momo*, *tsamtuk*, *thukpa*, and *suptuk*, a nettle soup). Because it is locally produced, rapeseed oil is almost the only vegetable oil available, and it is traditionally used for cooking meat and vegetables, or for frying bread.

Dairy products are generally integral part of the diet of mountain people. Nevertheless, the children’s consumption seems relatively low in the investigated areas. Less than 50% of the children drank milk during the day of recall. Furthermore, these figures include several children who only consumed small amounts of milk powder, which is used to make sweet tea. Dried cheese, which is called *chura kampo*, is made of dried curd [21]. It is also a very traditional food in rural Tibet. Very hard, it is chewed for hours as a snack. The consumption of other dairy products like yogurt and *chura loenpa*, a kind of cottage cheese, is anecdotal.

Very few vegetables enter the food consumption pattern of the rural children. Chinese cabbages (*Brassica rapa*, subspecies *pekinensis* and *chinensis*), Chinese radish (daikon), and green onions (scallions) are by far the most consumed vegetables. Wild nettles are sometimes eaten either fresh or dried in a traditional dish called *suptuk* or *satuk* [21,22], which has a consistency between soup and puree, depending on the addition of *tsampa* or not. It can include yak meat or fat and salt. However, the consumption of nettles tends to decrease since it is associated with a low socio-cultural level. Other vegetables such as tomato, cucumber, and zucchini are seldom consumed. Except for apples, eating fruits is also very uncommon in the investigated areas. The reason of the poor diversity of vegetables and fruit consumption probably lies in the harsh climate of the Himalayan plateau which does not allow their cultivation [1,4], and in the lack of incomes which prevents the rural people to buy imported foods. Several edible mushrooms are recognized by people [3], but few seem to have been consumed during the study, perhaps because it was not the season.

A new category of food products has appeared in the Tibetan food consumption pattern. Industrialized foods may be the flip side of the coin of the better accessibility to more diversified food products thanks to the infrastructural development. A relative high proportion of children consumed chips, candies, chocolate, biscuit, and cakes during the days of recall. It was also noticed that around 30% to 40% of the children ate instant noodles either as a meal or as a snack. This phenomenon rises some concerns because if industrialized foods—which are sometimes of poor nutritional value and rich in salt—are already a problem in many developed countries, they may have an even greater negative impact on populations with low incomes and low educational level like rural Tibetans [23,24]. 

### 3.2. Demographic Factors and Dietary Intakes

The descriptive statistics by counties are presented in Table 2. For both seasons (identified by the number following the variable: “1” for September 2012, and “2” for April 2013), the number of data (*n*), the mean value and the standard deviation are presented for several variables. They include: age, height, weight, energy, water and alcohol intakes, as well as the mean intakes of the investigated minerals. 

Table 3 shows the *p*-values of the ANOVA with two fixed factors. While few informative significant differences are highlighted for the season factor, two thirds of the variables present a *p*-value below 0.05 for the county factor. There are also significant interactions reflecting an interesting trend. The mean intakes of energy, water, and manganese tend to increase from September 2012 to April 2013 in Lhünzhub and Nyêmo, while it decreases in Maizhokungar. On the other hand, the mean intakes of potassium, magnesium, and selenium tend to decrease from September 2012 to April 2013 in Lhünzhub and Nyêmo, while it increases in Maizhokungar. A possible explanation to this tendency may lie in different dietary habits according to the season between the counties. 

In order to highlight the significant differences between the three counties, ANOVAs with one fixed factor (counties) were performed on every variable for both seasons, followed by multiple comparisons (results not shown). In September 2012, several significant differences were observed between Lhünzhub and Nyêmo counties for the following variables: energy, water, Mg, Fe, Zn, Mn, and Se. There is also a significant difference between Maizhokungar and Nyêmo counties for the weight, and the water, Zn, and Mn intakes. In April 2013, Cu and Se intakes differ significantly between Lhünzhub and Nyêmo counties. Energy and Se intakes differ significantly between Maizhokungar and Nyêmo counties. These recurrent differences between Nyêmo and the two others counties could find its roots in its location. Indeed, Lhünzhub and Maizhokungar are located at the north and northeast of Lhasa with relatively difficult access, while Nyêmo is at the southwest with easier access from Lhasa.

Even when significant differences are observed between Table 2 and Table 3, most of the intakes calculated by county and by season are far from the Chinese DRIs and the harmonized Estimated Average Requirements (hEARs). These hEARs are integrated to the IMAPP software and are derived from the recommended values of the most influent organizations (World Health Organization, Institute of Medicine of the U.S. National Academies of Sciences, etc.). Using the usual intakes calculated based on the whole dataset, i.e., including the three counties and both seasons, allows a more robust comparison. Table 4 presents the Chinese DRIs, the hEARs, the upper intake levels (UL), the usual intakes, the intakes at 5th and 95th percentiles, the percentage of inadequate intake, and the percentage of intakes above the UL. Except for Chinese DRIs, the other values have been obtained or computed via the IMAPP software [12]. All the children were considered as belonging to the category of age 4–8 years old. 

With 66% of the recommended nutrient intake (RNI), the usual intake of these Tibetan children is too low in calories. Although the energy required for growth is estimated to account for 3% of the total energy requirement [25], such low intakes may not be without consequences on the growth of these children. Not surprisingly, it has been reported that the weight-for-age and height-for-age P_50_ growth curves of Tibetan children are below those of the World Health Organization [7]. Another aspect of the energy requirement should be raised. Because people generally adapt their clothing and their environment to maintain thermoneutrality, there is no need of additional energy intake for thermoregulation [25]. However, it is possible that in the context of the very low temperatures of the Tibetan plateau and the scarce resources for heating and cooking, an increased need of energy for thermoregulation purposes becomes a relevant question. Another possible source of underestimation of the RNI is the physical activity level (PAL) of the children. Although it has neither been measured in this study, nor has it ever been reported to the best of our knowledge, several studies revealed a high exercise capacity of the Tibetan children [26]. Anyway, this energy deficit most likely results in underweight, growth retardation, and stunting, which are widely observed among Tibetan children [6,7,27].

No recommendation for water intakes is listed in the Chinese DRIs [11]. Harmonized EAR for total water is of 1.7 L/day for children aged 4–8 years. With a usual intake of 0.74 L/day which represents 45% of the adequate intakes (AI), more than 98% of the children present inadequate intakes. Even if the metabolism of the Tibetan population may be adapted to their harsh environment, a so low intake of water is susceptible, on the long term, to lead to severe dehydration with deleterious consequence on the metabolism. Traditionally, Tibetan peoples drink tea or homemade beer (*chang*) but little water. Until recently, the main sources of water for the population used to be well water or surface water [28], with all the risks related to chemical, microbial, and biological contaminants. During the study, only 5% and 12% of the children consumed river water (used in the preparation of tea drinks or soup) in September 2012 and April 2013, respectively. Most of the enrolled families have access to tap water which is expected to improve their water potability criteria. Hence, a higher consumption of water should be encouraged among the rural Tibetans.

Alcohol is known to have deleterious effects on adolescent’s brain [17,18]. In Western cultures, the age of first drinking in a parentally supervised environment is usually around 13 to 14 years old [29]. If it has been reported that 3–4 year old children could be able to recognize alcoholic drinks [30], it is uncommon to report alcohol consumption. With as much as 50% of the children having drinking alcohol in Nyêmo county during the recalls of April 2013, and a mean consumption of 6.8 g (Table 3), it cannot be without consequences on these children’s health. In addition to the adverse physical consequences of early alcohol consumption, there is a risk of a vicious circle involving an increase in consumption with age and a repeat of the educational scheme [29]. This is a potential public health problem that must be fought through education [31,32,33]. Although it has never been reported nor denied in T.A.R., alcohol consumption by pregnant mothers might also be a problem. Indeed, in utero alcohol exposure is susceptible to cause, inter alia, developmental abnormalities, growth retardation and abnormal gene expression [34]. The question of alcohol consumption by Tibetan women during pregnancy in T.A.R. deserves further attention.

Usual intake for sodium in this study represents 180% of the Chinese AI. No upper limit (UL) is entered in the Chinese table for sodium, while the Institute of Medicine set up the UL at 1900 mg/day for a 4–8 year child. More than 25% of the children exceed the UL, and the 95th percentile is above 3500 mg/day, which is susceptible to have deleterious consequences. Besides traditional foods, the high sodium intake comes mainly from two sources. The first is added salt. In the recalls of September 2012, 83% of the children had salt added in their meals, while, in April 2013, it was 95% of the children. The problem certainly comes from the amount of salt added. During the study, it was common to observe some families adding up to 20 to 30 g of salt in the cooking pot. Another important source of sodium arises from the consumption of instant noodles. As seen in Table 1, from 30% to 40% of the children consumed at least one serving size of instant noodles during the study, and it was not uncommon for some to have two. A classic serving size is about 77 g. According to the China FCT, it represents an intake of 880 mg of sodium, which consist in 98% of the Chinese AI. Overconsumption of sodium is a global health issue [35], and with his fast economic growth, its relentless urbanization, and a shift in its traditional diet, China is particularly exposed to this problem [36,37]. Experiencing the development of the modern infrastructures, with new access to industrial foods, combined with low education levels, the Tibetan populations are even more at risk and should raise the concerns of the competent authorities. Notwithstanding, thanks to the iodized salt program started in 1995 by the Chinese government, the majority of the rural families have access to this essential element for the growth that is iodine [38]. In the recalls of September 2012 and April 2013, 75% and 89%, respectively, of the families who added salt in their meals used iodized salt. Historically, rural people of T.A.R. trade lake salt with nomadic people for *tsampa*. Lake salt was still consumed by 14% of the children in the recall of September 2012, and by 15.6% in April 2013. In parallel to iodized salt, the consumption of lake salt may present some benefits. As it is unrefined, lake salt is richer in magnesium and in several trace elements, notably iron.

The usual intake for potassium in this study appears to only fulfill 60% of the Chinese RNI. However, the Chinese RNI for potassium only represents 40% of hEAR, and 99.9% of the children are having inadequate intakes. Severe potassium deficiency due to low dietary intakes in healthy subjects is rare [39], but it is increasingly evident that high sodium diet combined with low potassium intakes plays a major role in the pathogenesis of hypertension and cardiovascular diseases [40,41,42]. Although no ideal has yet been defined, recent studies suggest that a high sodium-to-potassium ratio is associated with significantly increased risk of cardiovascular diseases [43]. In the U.S. population, where heart disease is the leading cause of death [44], the median sodium-to-potassium ratio of suckling is below 1, and is increasing with age [45].The adult population (>20 y/o) presents an average ratio of 1.41 [46]. In this study the average ratio of the 3–5 years old children is 1.59. The Tibetans are no exception to the rule as it appears that more than 40% of the agricultural populations around Lhasa suffer from hypertension [47]. In addition to being a risk factor for cardiovascular disease, potassium deficiency is likely to increased bone turnover resulting in reduced bone formation and increased bone resorption [45]. The problem of imbalance in sodium and potassium intakes is exacerbated by the low level of awareness of the population. While the considerable sodium intakes come mainly from substantial amount of added salt and processed foods, the low intakes of potassium are a consequence of the low consumption of fruits and vegetables. As suggested by Xu et al. (2015), there are urgent needs for implementation of an efficient policy to control these risk factors [48]. A rapid preventive measure could be a modification of the iodized salt program in which the sodium chloride could be partially replaced by potassium chloride [49]. Nevertheless, on the long-term, an efficient policy, integrated in the cultural and socio-economic environment, must pass through the education of the population in order to rise their awareness and modify they dietary habits [47]. In our opinion, one of the most promising actions currently implemented by the Chinese government and some NGOs is the greenhouses construction, combined with seed banks [50,51]. This will allow the population to grow a wider variety of fruits and vegetables in a sustainable way, and so diversify their food intakes. 

With a usual intake representing less than 40% of Chinese adequate intake or the hEAR in calcium, and more than 90% of the children presenting inadequate intakes, the situation is challenging. Sufficient amount of this mineral is essential for a normal and healthy growth, and maintenance of the skeleton, especially during childhood [52]. Such long-term calcium deficiency inevitably leads to lower bone mineral content and bone mineral density, inducing stunting and even rickets, with higher risks to develop osteomalacia and osteoporosis in more advanced stage of life [39,53]. Although altitude is believed to play a role in growth retardation in Tibet [54,55,56], undernutrition unequivocally has its share of responsibility in stunted growth and signs of rickets which are widely observed in the Tibetan population [6,7,27,57]. The situation may be even worse if one take into account the relatively high intakes in phosphorus. Despite of little relevance in adults, the Ca:P ratio may be useful in assessing the quality of the diet in growing children [52]. According to the Chinese DRIs, the ideal Ca:P ratio (in mass) is 1.8. With a ratio of 0.55, calculated on the basis of the mean intakes, the balance is clearly unfavorable. It has to be reminded that the etiology of rickets is complex and not limited to calcium deficiency. There is more than one cause but it seems that all types of rickets share hypophosphatemia as a common symptom [58]. Hypophosphatemia in the Tibetan children could be, prima facie, surprising considering the more-than adequate intakes in phosphorus; 108% of the Chinese AI and 130% of hEAR. However, secondary hypophosphatemia may result from inadequate absorption of phosphorus due to a calcium deficient diet or because of vitamin D deficiency [59]. In the present case, it is demonstrated that the diet is poor in calcium. The vitamin D status of the Tibetans has been poorly studied and results are conflicting. Indeed, a study of Norsang et al. (2009) found that nomad people where predominantly deficient but farmers presented normal vitamin D serum levels [60]. On the contrary, analysis performed by the Kashin-Beck Disease Fund asbl-vzw revealed low vitamin D levels among farmers of the investigated area (unpublished results). Bioavailability of minerals is another factor that has to be taken into account in this problematic. The main source of calcium and phosphorus in the Tibetan diet is cereals and cereal products which contain substantial amounts of phytic acids [61]. During a preliminary study (unpublished data), we measured the phytic acid content in staples by a method adapted from Latta and Eskin (1980) [62]. The samples were collected in five families from Lhünzhub County and in five families from Nyêmo County. We found a mean phytic acid content of 372 ± 311 and 180 ± 50 mg/100 g (expressed as mean ± standard deviation, on a wet weight basis), for wheat flour (*n* = 19) and rice (*n* = 14), respectively. These results are in line with the values of Ma et al. (2005) [61]. The phytic acid content of *tsampa* (*n* = 20) was found to be much higher with a mean value of 1105 ± 177 mg/100 g. In another study, Ma et al. (2007) [63] concluded that the Chinese dietary intakes of phytate were high with risk of impairment of the bioavailability of calcium, iron, and zinc. It is highly probable that Tibetan people, with high consumption of tsampa, richer in phytic acid than wheat flour and rice, have higher phytate intakes and present even more unfavorable molar ratios of phytate-to-calcium, phytate-to-iron, phytate-to-zinc, and phytate × calcium/zinc. Several strategies are possible to remedy the situation. First of all it would be appropriate to confirm as accurately as possible the vitamin D status of the Tibetan population. Depending on the results, a supplementation in calcium and eventually in vitamin D would be a short-term solution. However, as Fang and Li (2014) [64] rightly recall: “Hereby, the dietary calcium deficiency, inadequate calcium intake, usually refers to failure to consume the full recommendation for calcium intake, which does not necessarily mean a true dietary deficiency for most of individuals”. The Tibetan diet is based on secular traditions and the people may have undergone physiological adaptation to low calcium intake. In this case, we have to be certain that long-term calcium supplement does not do more harm than good. It has recently been suggested that it may enhance the risk of cardiovascular disease [65,66], a factor that should not be added to a population already at risk without the certainty of beneficial health effects. If calcium deficiency should be confirmed by subsequent studies, a sustainable strategy could aim at decrease the phytic acid intake by decreasing the consumption of foods rich in phytates, and to diversify the dietary sources of calcium, which brings us back to support food diversification programs.

With its implication as a cofactor in more than 300 enzymatic reactions, and the storage of 50% to 60% of the body content into skeleton, magnesium is indubitably essential for normal growth and healthy bones [52]. Magnesium is probably not of concern, as the usual intake is ranging between the Chinese AI and the hEAR. Unfortunately, there is no consensus on how to evaluate the magnesium status. If severe deficiency can easily be detected, low chronic deficiency are much more difficult to characterize [39]. While the decrease in bioavailability of magnesium by phytic acid chelation is still controversial, the main source of dietary magnesium remains paradoxically whole cereal products, which are rich in phytate [67]. Besides, *tsampa* is richer in magnesium than wheat flour [14]. Other traditional Tibetan foods—such as dried cheese, dried wild peaches, dried nettles, and various dried leaves—are also rich in magnesium [14]. Regrettably, they are under consumed for cultural reasons, being associated with a low social level. The same discussion can apply for copper, for which the usual intake is slightly below the Chinese AI, and far from the hEAR. Copper is also essential for bone and joints growth [10], and its decreased bioavailability due to phytic acid is as well controversial [68]. High zinc and iron intakes may decrease copper absorption [39], but it is unlikely in the diet of the enrolled children considering the present results. Alcohol has also been reported to decrease copper absorption, which could be a problem for some children who present high *chang* consumption [39]. Investigating on biological markers would be advisable in order to conclude about the copper status of the Tibetan children [69].

Iron usual intake is very close to the Chinese AI. However, such DRI is made assuming a balanced intake between heme iron and non-heme iron. In a western diet—in which approximately 75% of iron is supposed from heme iron sources—it is estimated that approximately 18% of the total ingested iron is absorbed, while it decreases to 5%–10% in a vegetarian diet [70]. Adequate iron intake in the case of vegetarian diet is therefore recommended to be doubled. With a low consumption of meat products, the Tibetan diet is closer to the vegetarian diet. The major source of dietary iron in the Tibetan diet is the cereal products, including *tsampa*, wheat flour, and rice. Phytic acid is well known to decrease the bioavailability of iron [71]. Tea drinks—including yak butter tea, black tea, and sweet tea—are abundantly consumed and even mixed with the flours and are also known to negatively affect iron absorption due to their polyphenol and tannin contents [39]. On the other hand, some components known to enhance iron absorption, such as ascorbic acid, are not much present in the Tibetan diet. Investigating the iron status of the Tibetan population would also deserve some attention, especially since signs of anemia have already been observed [27,72]. Iron is also essential to bone integrity [10], and we can wonder if a strong mobilization of the body iron pool in order to maintain a sufficient hemoglobin concentration in a high altitude context could cause deleterious effects on bone health.

If iron intakes may deserve further investigation because of a possible low bioavailability, zinc intakes are much more debatable. Indeed, zinc has also its bioavailability strongly affected by foods high in phytic acid [71], but the usual intake calculated in this study provides only 36% of the Chinese RNI. The recommended dietary allowance (RDA) of the U.S. institute of Medicine for zinc is 5 mg/day for the same category of age (which represent 40% of the Chinese RNI), and the hEAR is about 4 mg/day. This discrepancy probably arises from the difference in food consumption pattern between western and eastern diets, the first being richer in meat products. It is in accordance with the suggestion of a two-fold greater RDA in the case of a vegetarian diet [70]. It is important to consider that zinc recommendations are formulated as recommended nutrient intake (RNIs) and not adequate intakes (AIs). Contrary to AIs which are not based on sufficient scientific evidences, RNIs can be associated to the RDAs and are calculated based on estimated average requirement so that the risk of inadequacy to an individual is very small [70]. With a usual intake of 4.3 mg/day, and cereals products as the main source of dietary zinc, it would not be surprising that the majority of children have a zinc deficiency.

An ideal solution to the problem of low intakes in iron, zinc, and copper would be a higher consumption of meat and fish. However, for economic and philosophical reasons, or because of lack of availability, this solution is hardly possible. Fishes are scarce in many rural areas and are never consumed by local people for cultural reasons. If yak, sheep and goat are raised, they are very useful in the traditional rural life and hence sparingly butchered. Poultry breeding could be a solution to improve the supply of meat, but the Tibetans prefer to eat large animals in order to sacrifice the least possible lives [21]. The next best alternative to our opinion has already been mentioned and consists in food diversification. Greenhouses could allow people to growth vegetables rich in iron such as parsley, which is by the way rich in vitamin C, an enhancer of iron absorption [70]. Lentils or beans could also be a valuable source of iron and zinc [73]. Some genotypes present relatively low levels of phytic acid, and germination or fermentation could further increase the bioavailability of these minerals [74,75]. Nettles as well as many other wild dried herbs and dried mushrooms also have high iron and zinc contents [3,14]. Their consumption should be encouraged despite their difficult acceptation by local people, at least as regards the nettles. Anecdotally eaten as a snack by some children, sunflower seeds are also rich in iron and zinc. Cultivation of *Helianthus* and promotion of seeds consumption would be for sure beneficial. Poultry breeding for egg consumption could also be a possible way to improve the overall quality of the diet. 

No recommendation for manganese is provided in the Chinese DRIs [11]. Compared to hEAR the usual intake calculated in this study represents more than 180% of the recommendation, and almost 20% of the children are above the UL, set up at 3 mg/day. These high intakes may not be of concern because manganese comes primarily from cereal products for which the intestinal absorption rate is generally low. However, some children have a high consumption of tea drinks and *chang*, relatively rich in Mn, with a higher bioavailability [14,39]. If the high intake is recurring, it could be neurotoxic for these children [76].

The usual selenium intake fulfills less than 45% of the Chinese RNI, and it is probably overestimated. Many vegetables consumed by Tibetans are produced locally but have not been analyzed for their selenium content. The values of Chinese FCT which were used are likely to be overestimated because of the very low level of selenium in the soils of these regions [8,77]. One example is the Chinese cabbage for which several samples were analyzed in a previous study and whose selenium concentrations were below 0.01 µg/100 g while the value found in the Chinese FCT is about 0.49 µg/100 g [11,78]. It is worth emphasizing that selenium recommendations are also expressed as RNIs and not AIs, underlying the importance to meet the daily requirements. Selenium is an essential trace element whose deprivation can have many deleterious health effects [39]. With regard to bone and joints metabolisms, selenium deficiency has been reported to induce growth retardation, osteopenia, and chondronecrosis in animal and in vitro models [79,80]. Selenium deficiency has been clearly observed among the Tibetan populations living in areas endemic for the Kashin-Beck disease [81,82,83]. If the imbalances in the previous minerals could be relatively easily solved through food diversification, the selenium issue is much more challenging. Indeed, selenium dietary deficiency is rooted in a serious deficiency of the soil, and the solution lies in the import of selenium in one form or another. Several studies have been conducted to investigate the influence of the consumption of selenium-enriched salt on the prevalence of Kashin-Beck disease [84,85]. Beneficial effects have been observed, if not always on the KBD prevalence, at least on the selenium status of the people [86,87,88]. However, it is clearly not a long-term sustainable solution [89], and its implementation on a large scale would be difficult. Besides, given the amounts of salt consumed by the Tibetans, there could also be a risk of selenium overconsumption with an underlying risk of toxicity [90]. Infrastructure development allows Tibetans to have access to foods rich in selenium coming from other region of China. Positive effects were observed on the selenium status [91], but it is not an economically viable solution, especially for the poorest rural people, who are the most affected [92]. Selenium-supplemented fertilizer has also been used in some provinces of China endemic for KBD, and it has been shown to increase the average Se content in total diets [93]. The Se-supplemented fertilizers could also have benefits on the crop yields and animal nutrition [94]. This solution has already been implemented at a nation-wide level in Finland and has been proved to be an effective, safe and controlled way of bringing the selenium intake of the population to the recommended level [95]. It is perhaps the most appropriate solution to the selenium issue in T.A.R. and in the other soil-selenium deficient areas in China. 

## 4. Conclusions

Clinical signs of stunted growth and rickets have frequently been reported among rural populations of T.A.R., and especially amid children. Further, a chronic and endemic osteochondropathy called Kashin-Beck disease plagues these rural populations, although its overall prevalence seems to decrease for some time. A healthy bone metabolism depends on many factors, including balanced intakes in several minerals. This study aimed at calculates the usual intakes of rural Tibetan children for the main elements of dietary relevance. Further studies are mandatory to confirm the results and the trends revealed by the present study. However, in the investigated areas, the diet of the children appears to be very monotonous and poorly diversified. It is mainly based on cereals products, with low consumption of meat and dairy products, and even lower consumption of vegetables and fruits. Local alcohol drinking is not uncommon even in young children. This diet presents several imbalances in mineral intakes with possible consequences on growth and bone metabolism of the children. Sodium intakes are excessive, while there are very low for K, Ca, Zn, Cu, and Se. Bioavailability of Ca, Fe, and Zn may be of concern due to the high phytic acid content in the diet. Experiencing fast development of its infrastructures, T.A.R. is changing. Although still very traditional, these changes are beginning to influence eating habits of rural people, with positive and negative effects. A better accessibility to food products that cannot be grown at such an altitude—like rice, vegetables, and fruits—has a beneficial impact on the diversification of the diet. On the other hand, industrialized foods and beverages of limited nutritional quality are frequently consumed. However, rural people, with frequent low education levels, are not aware of the potential risks of non-moderate consumption of these foods. Several solutions can be envisaged to improve the situation, and some are already implemented. An elegant one is the promotion of food diversification programs and education programs to raise awareness among Tibetans about healthy eating practices. The distribution of selenium-supplemented fertilizers, and in the most urgent situations, the distribution of nutritional supplements also deserves consideration. The ideal solution should definitely be integrated, sustainable and fully adapted to the socio-cultural environment of T.A.R. 

## Figures and Tables

**Table 1:** Relative consumption (%) of different foodstuffs by young rural Tibetan children according to the factors county and season.

	September 2012			April 2013		
	L (*n* = 70)	M (*n* = 27)	N (*n* = 153)	L (*n* = 70)	M (*n* = 27)	N (*n* = 153)
**Beverages**						
Butter tea	69	93	82	83	96	82
Black tea	16	4	8	36	11	12
Sweet tea	1	0	16	7	15	12
Chang (local beer)	16	4	34	14	4	52
Various soft drink	24	56	15	37	26	10
**Staples**						
Tsampa	66	78	80	74	85	78
Rice	50	52	56	50	59	57
Wheat flour	29	33	41	67	22	42
Wheat noodle (fresh or dried)	6	15	13	13	22	15
Potato	33	37	59	43	37	58
**Meat**						
Dried yak	5.7	3.7	2.0	7.1	7.4	9.2
Fresh yak/beef	21	37	11	43	41	20
Mutton/goat	1.4	7.4	24	2.9	0.0	13
Pork	10	11	11	11	11	7.8
Chicken	2.9	0.0	2.0	4.3	3.7	2.0
Egg	5.7	15	9.2	2.9	3.7	6.5
**Fats**						
Yak fat	11	7	23	20	19	29
Butter	77	93	86	89	96	84
Margarin	4	11	5	36	56	42
Rapeseed oil	46	56	50	59	37	58
**Dairy products**						
Milk (fresh or powder)	53	44	42	40	41	41
Dried cheese	20	56	48	53	59	29
Cottage cheese	1.4	3.7	3.9	1.4	0.0	5.9
Yogurt	13	33	6.5	1.4	11	3.9
**Vegetables**						
Cabbages (all included)	19	33	17	23	3.7	17
Chinese radish (daikon)	10	15	25	20	19	26
Onion (scallion)	11	11	8.5	10	11	15
Green chili	0.0	0.0	3.9	0.0	0.0	0.7
Tomato	0.0	7.4	1.3	0.0	0.0	0.0
Cucumber	7.1	3.7	0.0	0.0	0.0	0.0
Zucchini	4.3	3.7	0.7	0.0	3.7	0.0
Spinach	1.4	0.0	0.7	1.4	0.0	0.7
Cauliflower	1.4	0.0	0.7	1.4	0.0	0.7
Carrot	0.0	0.0	0.7	0.0	3.7	2.0
Asparagus lettuce	2.9	0.0	0.0	1.4	7.4	0.7
Nettles	4.3	0.0	0.7	14.3	0.0	2.6
Mushroom	0.0	0.0	0.7	0.0	7.4	0.0
**Fruits**						
Apple	17	11	13	13	0	8.5
Peach (fresh or dried)	1.4	0.0	11	2.9	0.0	2.0
Orange	0.0	0.0	0.7	0.0	0.0	0.0
Pear	0.0	0.0	0.0	0.0	0.0	1.3
Hawthorn	1.4	0.0	0.0	0.0	0.0	0.0
Watermelon	0.0	3.7	0.0	0.0	0.0	0.0
Banana	1.4	7.4	0.7	4.3	7.4	0.7
Plum	0.0	0.0	0.0	1.4	0.0	2.0
**Various**						
Salt (added)	71	85	88	97	89	95
MSG (sodium glutamate)	5.7	7.4	29	5.7	3.7	37
Instant noodle	30	41	37	37	30	37
Chips	0.0	0.0	0.7	1.4	3.7	1.3
Mixed candy	33	15	21	54	26	35
Chocolate	0.0	0.0	2.0	1.4	11	0.0
Biscuit and cake	21	15	14	24	19	28

The relative consumption is calculated based on the number of children who consumed a given foodstuff divided by the total number of children enrolled in the county, expressed in percentage. The letters L, M, and N stand for Lhunzhub, Maizhokungar, and Nyêmo respectively.

**Table 2:** Demographic factors and dietary intakes by county and by season.

		Lhünzhub (*n* = 70)		Maizhokungar (*n* = 27)		Nyêmo (*n* = 153)	
	Season	Mean	SD	Mean	SD	Mean	SD
Age	1 ^a^	4.0	0.8	4.0	0.7	4.0	0.8
	2	4.5	0.7	4.6	0.8	4.8	0.9
Weight	1 ^b^	14.3	2.6	15.1	2.5	13.6	2.5
	2	16.4	3.6	16.5	2.6	15.4	2.6
Height	1 ^b^	97	9	94	9	95	8
	2 ^c^	103	10	102	7	102	8
Energy	1	4289	1790	4157	2236	3546	1902
	2	4490	2126	3309	1336	3996	1842
Water	1	811	386	798	334	606	354
	2	857	397	705	354	792	350
Alcohol	1 ^d^	2.6	1.5	1.5	-	1.9	1.9
	2 ^e^	3.5	2.7	4.4	-	6.8	5.0
Na	1	1945	1152	1739	944	1580	1327
	2	1838	1366	1361	818	1432	1337
K	1	998	571	981	511	821	533
	2	933	436	813	552	936	497
Ca	1	361	314	394	252	251	374
	2	330	276	266	158	269	235
P	1	575	306	588	272	487	387
	2	564	295	533	348	555	314
Mg	1	157	71	143	72	116	57
	2	156	70	130	89	154	75
Fe	1	14.7	10.1	12.0	6.0	11.2	10.4
	2	13.7	6.2	10.5	7.0	12.1	7.0
Zn	1	4.9	2.2	5.2	4.2	3.6	2.2
	2	5.0	2.5	4.7	3.4	4.2	2.1
Cu	1	0.67	0.28	0.72	0.37	0.79	1.10
	2	0.77	0.47	0.80	0.98	1.14	0.75
Mn	1	2.47	1.06	2.29	1.04	1.68	0.70
	2	2.85	1.50	2.00	1.06	2.29	1.08
Se	1	12.5	8.2	13.6	12.2	9.1	9.3
	2	12.3	8.8	8.9	7.2	10.6	10.0

Season 1 corresponds to September 2012, Season 2 corresponds to April 2013. SD = standard deviation. ^a^ *n* = 152 for Nyêmo; ^b^ *n* = 148 for Nyêmo; ^c^ *n* = 69 for Lhünzhub; ^d^ *n* = 11, 1, and 52 for Lhünzhub, Maizhokungar, and Nyêmo, respectively; ^e^ *n* = 10, 1, and 80 for Lhünzhub, Maizhokungar, and Nyêmo, respectively Age is expressed in year, weight in kg, height in cm, energy in kJ, water and alcohol in g, and all elements in mg, except selenium which is expressed in µg.

**Table 3:** *p*-values of ANOVA with two fixed factors, counties (3 levels), and seasons (2 levels).

	Season	County	Season * County
Age (y)	<0.001	0.265	<0.001
Weight (kg)	<0.001	0.005	0.442
Height (cm)	<0.001	0.449	0.720
Energy (kJ)	0.735	0.011	0.038
Water (g)	0.224	0.004	0.007
Na (mg)	0.107	0.024	0.753
K (mg)	0.430	0.339	0.030
Ca (mg)	0.138	0.021	0.187
P (mg)	0.984	0.383	0.241
Mg (mg)	0.269	0.022	0.001
Fe (mg)	0.576	0.018	0.365
Cu (mg)	0.042	0.013	0.202
Zn (mg)	0.823	<0.001	0.117
Mn (mg)	0.036	<0.001	0.006
Se (µg)	0.227	0.052	0.044

**Table 4:** Chinese DRIs (China CDC, 2009), hEARs, ULs, usual intakes, and risks of inadequate intakes of the rural Tibetan children (*n* = 250, mean for: age = 4.4 y.; weight = 14.9 kg; height = 99 cm).

	Chinese AI/*RNI*	hEAR	UL	Usual Intake	Intake 5th	Intake 95th	% of Inadequacy	% > UL
Energy (kcal)	*1430* *	ND	ND	943	389	1795	ND	ND
Water (g)	-	1700	ND	742	229	1436	98.3	ND
Alcohol (g)	-	ND	ND	4.6 ^†^	ND	ND	ND	ND
Na (mg)	900	900	1900	1619	375	3565	26.3	27.6
K (mg)	1500	3800	ND	910	310	1880	99.9	ND
Ca (mg)	800	640	2500	292	48.7	784	92,0	0.2
P (mg)	500	405	3000	539	197	1145	40.7	0,0
Mg (mg)	150	110	ND	141	58.6	278	39.5	ND
Fe (mg)	12	ND	40	12.3	4.6	26.8	ND	0,0
Zn (mg)	*12*	4.0	12	4.3	1.6	9	55.5	1.5
Cu (mg)	1.00	3.4	30	0.87	ND	ND	ND	ND
Mn (mg)	-	1.2	3	2.19	0.87	4.31	15.9	18.8
Se (µg)	*25*	23	150	10.72	ND	ND	ND	ND

Chinese recommended nutrient intakes (RNIs) are in italic type and adequate intakes (AIs) are in ordinary type. * RNI for energy is the mean RNI for children from 3 to 5 years old, both gender included. Harmonized Estimated Average Requirements (hEAR), Upper intake levels (UL), Usual intakes, Intakes at 5th and 95th percentiles, and risks of inadequate intakes have been computed with IMAPP software. ND = not determined. ^†^ Consumption data for alcohol correspond to mean intake and not usual intakes.

## References

[1] Malaisse F., Lacroix D., Bock L., Haubruge E., Chasseur C., Malaisse F., Mathieu F. (2008). Framework. Big Bone Disease. A Multidisciplinary Approach of Kashin-Beck Disease in Tibet Autonomous Region (P.R. China).

[2] Rooze S., de Voghel P., Mathieu F., Robert M., Lobsang R., Wangdu L., Dermience M., Lognay G., Goyens P. Food intake of Tibetan children living in Kashin Beck disease endemic areas in Central Tibet. Proceedings of the BIT’s 1st Annual Congress of Nutrition & Health 2013 (WCNH-2013).

[3] Malaisse F., Lognay G., Haubruge E., De Kesel A., Delcarte E., Wathelet B., Malaisse F., Mathieu F. (2008). The alternative food path or the very little diversified diet hypothesis. Big Bone Disease. A Multidisciplinary Approach of Kashin-Beck Disease in Tibet Autonomous Region (P.R. China).

[4] Chasseur C., Malaisse F., Haubruge E., Wangla R., Suetens C., Malaisse F., Mathieu F. (2008). The land-ecosystem. Big Bone Disease. A Multidisciplinary Approach of Kashin-Beck Disease in Tibet Autonomous Region (P.R. China).

[5] Goyens P., Bally P., Claus W., Yangzom D., Mathieu F., Malaisse F., Mathieu F. (2008). Nutritional issues in KBD endemic rural areas. Big Bone Disease. A Multidisciplinary Approach of Kashin-Beck Disease in Tibet Autonomous Region (P.R. China).

[6] Harris N.S., Crawford P.B., Yangzom Y., Pinzo L., Gyaltsen P., Hudes M. (2001). Nutritional and Health Status of Tibetan Children Living at High Altitudes. N. Engl. J. Med..

[7] Rooze S., Dramaix-Wilmet M., Mathieu F., Bally P., Yangzom D., Li J., Goyens P. (2012). Growth, nutritional status, and signs of rickets in 0–5-year-old children in a Kashin-Beck disease endemic area of Central Tibet. Eur. J. Pediatr..

[8] Chasseur C., Suetens C., Begaux F., Haubruge E., Wangla R., Rinchen L., Wangdu L., Claus W., Mathieu F., Malaisse F., Mathieu F. (2008). The mineral defiiency hypothesis. Big Bone Disease. A Multidisciplinary Approach of Kashin-Beck Disease in Tibet Autonomous Region (P.R. China).

[9] Wang Z., Dang S., Yan H. (2010). Nutrient intakes of rural Tibetan mothers: A cross-sectional survey. BMC Public Health.

[10] Dermience M., Lognay G., Mathieu F., Goyens P. (2015). Effects of thirty elements on bone metabolism. J. Trace Elem. Med. Biol..

[11] Chinese Center for Disease Control and Prevention (2009). China Food Composition.

[12] Carriquiry A., Murphy S., Allen L., de Benoit B., Rogers L. (1999). IMAPP (Intake Modeling, Assessment and Planning Program).

[13] Dermience M., Mathieu F., Barthélemy J.P., Maesen P., Romnee J.M., De Maertelaer V., Yangzom D., Tsewang P., Lognay G. (2013). The relevance of food composition data for nutrition surveys in rural Tibet: Pilot study in the context of Kashin-Beck Disease. Biotechnol. Agron. Société Environ..

[14] Dermience M., Li X.W., Mathieu F., Claus W., De Maertelaer V., Yangzom D., Lognay G. (2014). Minerals and trace elements in traditional foods of rural areas of Lhasa Prefecture, Tibet Autonomous Region (P.R. China). J. Food Compos. Anal..

[15] US Departement of Agriculture (2010). USDA Food search for Windows.

[16] Gibson R.S., Ferguson E.L. An Interactive 24-Hour Recall for Assessing the Adequacy of Iron and Zinc Intakes in Developing Countries. https://assets.publishing.service.gov.uk/media/57a08bac40f0b64974000cd6/tech08.pdf.

[17] Jacobus J., Tapert S.F. (2013). Neurotoxic effects of alcohol in adolescence. Annu. Rev. Clin. Psychol..

[18] (2004). Hiller-Sturmhöfel; Swartzwelder Alcohol’s Effects on the Adolescent Brain. Alcohol Res. Health.

[19] Popkin B.M. (1999). Urbanization, Lifestyle Changes and the Nutrition Transition. World Dev..

[20] Haubruge E., Chasseur C., Debouck C., Begaux F., Suetens C., Mathieu F., Michel V., Gaspar C., Rooze M., Hinsenkamp M., Gillet P. (2001). The prevalence of mycotoxins in Kashin-Beck disease. Int. Orthop..

[21] Dorje R. (1985). Food in Tibetan Life.

[22] Malaisse F., Mathieu F., Delcarte E., Wathelet B., Claus W., Goyens P., Malaisse F., Mathieu F. (2008). Protinet. Big Bone Disease. A Multidisciplinary Approach of Kashin-Beck Disease in Tibet Autonomous Region (P.R. China).

[23] Du S., Mroz T.A., Zhai F., Popkin B.M. (2004). Rapid income growth adversely affects diet quality in China—Particularly for the poor!. Soc. Sci. Med. 1982.

[24] Tzioumis E., Adair L.S. (2014). Childhood dual burden of under-and overnutrition in low-and middle-income countries: A critical review. Food Nutr. Bull..

[25] Institute of Medicine, Food and Nutrition Board (2005). Dietary Reference Intakes for Energy, Carbohydrate, Fiber, Fat, Fatty Acids, Cholesterol, Protein, and Amino Acids (Macronutrients).

[26] Bianba, Berntsen S., Andersen L.B., Stigum H., Ouzhuluobu, Nafstad P., Wu T., Bjertness E. (2014). Exercise Capacity and Selected Physiological Factors by Ancestry and Residential Altitude: Cross-Sectional Studies of 9–10-Year-Old Children in Tibet. High Alt. Med. Biol..

[27] Dang S., Yan H., Yamamoto S., Wang X., Zeng L. (2004). Poor nutritional status of younger Tibetan children living at high altitudes. Eur. J. Clin. Nutr..

[28] Zhang B., Yang L., Wang W., Li Y., Li H. (2011). Environmental selenium in the Kaschin-Beck disease area, Tibetan Plateau, China. Environ. Geochem. Health.

[29] Newbury-Birch D., Gilvarry E., McCardle P., Ramesh V., Stewart S., Walker J., Avery L., Beyer F., Brown N., Jackson K. (2009). Impact of Alcohol Consumption on Young People a Systematic Review of Published Reviews.

[30] Lang A.R., Stritzke W.G. (1993). Children and alcohol. Recent Dev. Alcohol..

[31] Guo W., Lanzi G., Luobu O., Ma X., Zhen P., Ji Y., Wei G., Wang Z., Deng W., Zhuoma B. (2008). An epidemiological survey of alcohol use disorders in a Tibetan population. Psychiatry Res..

[32] Schnohr C., Højbjerre L., Riegels M., Ledet L., Larsen T., Schultz-Larsen K., Petersen L., Prescott E., Grønbaek M. (2004). Does educational level influence the effects of smoking, alcohol, physical activity, and obesity on mortality? A prospective population study. Scand. J. Public Health.

[33] Tang Y., Xiang X., Wang X., Cubells J.F., Babor T.F., Hao W. (2013). Alcohol and alcohol-related harm in China: Policy changes needed. Bull. World Health Organ..

[34] Abbott C.W., Kozanian O.O., Kanaan J., Wendel K.M., Huffman K.J. (2016). The Impact of Prenatal Ethanol Exposure on Neuroanatomical and Behavioral Development in Mice. Alcohol. Clin. Exp. Res..

[35] World Health Organization (2007). Reducing Salt Intake in Populations: Report of a WHO Forum and Technical Meeting.

[36] Zhai F.Y., Du S.F., Wang Z.H., Zhang J.G., Du W.W., Popkin B.M. (2014). Dynamics of the Chinese diet and the role of urbanicity, 1991–2011. Obes. Rev..

[37] Zhang R., Wang Z., Fei Y., Zhou B., Zheng S., Wang L., Huang L., Jiang S., Liu Z., Jiang J. (2015). The Difference in Nutrient Intakes between Chinese and Mediterranean, Japanese and American Diets. Nutrients.

[38] Lu A. Iodized Salt Distributed to All Rural Townhips in Tibet. http://www.gov.cn/english/2012–02/03/content_2057954.htm.

[39] Martin A. (2000). Apports Nutritionnels Conseillés Pour la Population Française.

[40] Aaron K.J., Sanders P.W. (2013). Role of dietary salt and potassium intake in cardiovascular health and disease: A review of the evidence. Mayo Clin. Proc..

[41] Adrogué H.J., Madias N.E. (2014). The impact of sodium and potassium on hypertension risk. Semin. Nephrol..

[42] Adrogué H.J., Madias N.E. (2007). Sodium and potassium in the pathogenesis of hypertension. N. Engl. J. Med..

[43] Yang Q., Zhang Z., Kuklina E.V., Fang J., Ayala C., Hong Y., Loustalot F., Dai S., Gunn J.P., Tian N. (2012). Sodium Intake and Blood Pressure Among U.S. Children and Adolescents. Pediatrics.

[44] U.S. Center for Disease Control and Prevention Heart Disease Facts & Statistics | cdc.gov. http://www.cdc.gov/heartdisease/facts.htm.

[45] Institute of Medicine of The National Academies (2005). Dietary Reference Intakes for Water, Potassium, Sodium, Chloride, and Sulfate.

[46] Zhang Z., Cogswell M.E., Gillespie C., Fang J., Loustalot F., Dai S., Carriquiry A.L., Kuklina E.V., Hong Y., Merritt R. (2013). Association between usual sodium and potassium intake and blood pressure and hypertension among U.S. adults: NHANES 2005–2010. PLoS ONE.

[47] Yao D.-K., Su W., Zheng X., Wang L.-X. (2016). Knowledge and Understanding of Hypertension Among Tibetan People in Lhasa, Tibet. Heart Lung Circ..

[48] Xu S., Jiayong Z., Li B., Zhu H., Chang H., Shi W., Gao Z., Ning X., Wang J. (2015). Prevalence and Clustering of Cardiovascular Disease Risk Factors among Tibetan Adults in China: A Population-Based Study. PLoS ONE.

[49] Levings J.L., Gunn J.P. (2014). The Imbalance of Sodium and Potassium Intake: Implications for Dietetic Practice. J. Acad. Nutr. Diet..

[50] China Daily USA Greenhouses Help Grow Family Incomes. http://usa.chinadaily.com.cn/epaper/2015-06/05/content_20918649.htm.

[51] KBD Fund Projects—Kashin-Beck Disease Fund asbl-vzw. http://www.kbdfund.org/projects.html.

[52] Institute of Medicine (1997). Dietary Reference Intakes for Calcium, Phosphorus, Magnesium, Vitamin D, and Fluoride.

[53] Marieb E.N. (2005). Anatomie et Physiologies Humaines.

[54] Argnani L., Cogo A., Gualdi-Russo E. (2008). Growth and nutritional status of Tibetan children at high altitude. Coll. Antropol..

[55] Bianba B., Yangzong Y., Gonggalanzi G., Berntsen S., Andersen L.B., Stigum H., Nafstad P., Bjertness E. (2015). Anthropometric Measures of 9-to 10-Year-Old Native Tibetan Children Living at 3700 and 4300 m Above Sea Level and Han Chinese Living at 3700 m. Medicine (Baltimore).

[56] Dang S., Yan H., Yamamoto S. (2008). High altitude and early childhood growth retardation: New evidence from Tibet. Eur. J. Clin. Nutr..

[57] Dang S., Yan H., Wang D. (2014). Implication of World Health Organization growth standards on estimation of malnutrition in young Chinese children: Two examples from rural western China and the Tibet region. J. Child Health Care.

[58] Tiosano D., Hochberg Z. (2009). ’ev Hypophosphatemia: The common denominator of all rickets. J. Bone Miner. Metab..

[59] Allgrove J., Mughal M.Z. (2014). Calcium deficiency rickets: Extending the spectrum of “nutritional” rickets. Arch. Dis. Child..

[60] Norsang G., Ma L., Dahlback A., Zhuoma C., Tsoja W., Porojnicu A., Lagunova Z., Moan J. (2009). The Vitamin D Status Among Tibetans. Photochem. Photobiol..

[61] Ma G., Jin Y., Piao J., Kok F., Guusje B., Jacobsen E. (2005). Phytate, Calcium, Iron, and Zinc Contents and Their Molar Ratios in Foods Commonly Consumed in China. J. Agric. Food Chem..

[62] Latta M., Eskin M. (1980). A simple and rapid colorimetric method for phytate determination. J. Agric. Food Chem..

[63] Ma G., Li Y., Jin Y., Zhai F., Kok F.J., Yang X. (2007). Phytate intake and molar ratios of phytate to zinc, iron and calcium in the diets of people in China. Eur. J. Clin. Nutr..

[64] Fang A., Li K. (2014). Calcium deficiency: Where does the diagnostic criterion come from and by what is bone health influenced?. Chin. Med. J. (Engl.).

[65] Daly R.M., Ebeling P.R. (2010). Is Excess Calcium Harmful to Health?. Nutrients.

[66] Reid I.R. (2013). Cardiovascular effects of calcium supplements. Nutrients.

[67] Bertinato J., Plouffe L.J., Lavergne C., Ly C. (2014). Bioavailability of magnesium from inorganic and organic compounds is similar in rats fed a high phytic acid diet. Magnes. Res..

[68] Lopez H.W., Leenhardt F., Coudray C., Remesy C. (2002). Minerals and phytic acid interactions: Is it a real problem for human nutrition?. Int. J. Food Sci. Technol..

[69] Olivares M., Méndez M.A., Astudillo P.A., Pizarro F. (2008). Present situation of biomarkers for copper status. Am. J. Clin. Nutr..

[70] Institute of Medicine, Food and Nutrition Board (2001). Dietary Reference Intakes for Vitamin A, Vitamin K, Arsenic, Boron, Chromium, Copper, Iodine, Iron, Manganese, Molybdenum, Nickel, Silicon, Vanadium, and Zinc.

[71] Kumar V., Sinha A.K., Makkar H.P.S., Becker K. (2010). Dietary roles of phytate and phytase in human nutrition: A review. Food Chem..

[72] Xing Y., Yan H., Dang S., Zhuoma B., Zhou X., Wang D. (2009). Hemoglobin levels and anemia evaluation during pregnancy in the highlands of Tibet: A hospital-based study. BMC Public Health.

[73] Thavarajah D., Thavarajah P., Sarker A., Vandenberg A. (2009). Lentils (Lens culinaris Medikus Subspecies culinaris): A whole food for increased iron and zinc intake. J. Agric. Food Chem..

[74] Ayet G., Burbano C., Cuadrado C., Pedrosa M.M., Robredo L.M., Muzquiz M., de la Cuadra C., Castaño A., Osagie A. (1997). Effect of Germination, under Different Environmental Conditions, on Saponins, Phytic Acid and Tannins in Lentils (*Lens culinaris*). J. Sci. Food Agric..

[75] Thavarajah P., Thavarajah D., Vandenberg A. (2009). Low phytic acid lentils (*Lens culinaris* L.): A potential solution for increased micronutrient bioavailability. J. Agric. Food Chem..

[76] Aschner J.L., Aschner M. (2005). Nutritional aspects of manganese homeostasis. Mol. Aspects Med..

[77] Li S., Li W., Hu X., Yang L., Xirao R. (2009). Soil selenium concentration and Kashin-Beck disease prevalence in Tibet, China. Front. Environ. Sci. Eng. China.

[78] Dermience M. (2010). Kashin-Beck Disease: Evaluation of Mineral Intake in Young Tibetan Children from Endemic Areas. Bioengineer Master’s Thesis.

[79] Moreno-Reyes R., Egrise D., Nève J., Pasteels J.-L., Schoutens A. (2001). Selenium Deficiency-Induced Growth Retardation Is Associated with an Impaired Bone Metabolism and Osteopenia. J. Bone Miner. Res..

[80] Ren F.L., Guo X., Zhang R.J., Wang S.J., Zuo H., Zhang Z.T., Geng D., Yu Y., Su M. (2007). Effects of selenium and iodine deficiency on bone, cartilage growth plate and chondrocyte differentiation in two generations of rats. Osteoarthr. Cartil..

[81] Moreno-Reyes R., Suetens C., Mathieu F., Begaux F., Zhu D., Rivera M.T., Boelaert M., Nève J., Perlmutter N., Vanderpas J. (1998). Kashin-Beck Osteoarthropathy in Rural Tibet in Relation to Selenium and Iodine Status. N. Engl. J. Med..

[82] Yang L., Zhao G.-H., Yu F.-F., Zhang R.-Q., Guo X. (2016). Selenium and Iodine Levels in Subjects with Kashin-Beck Disease: A Meta-analysis. Biol. Trace Elem. Res..

[83] Yao Y., Pei F., Kang P. (2011). Selenium, iodine, and the relation with Kashin-Beck disease. Nutrition.

[84] Jirong Y., Huiyun P., Zhongzhe Y., Birong D., Weimin L., Ming Y., Yi S. (2012). Sodium selenite for treatment of Kashin-Beck disease in children: A systematic review of randomised controlled trials. Osteoarthr. Cartil..

[85] Yu F., Han J., Wang X., Fang H., Liu H., Guo X. (2016). Salt-Rich Selenium for Prevention and Control Children with Kashin-Beck Disease: A Meta-analysis of Community-Based Trial. Biol. Trace Elem. Res..

[86] Moreno-Reyes R., Mathieu F., Boelaert M., Begaux F., Suetens C., Rivera M.T., Neve J., Perlmutter N., Vanderpas J. (2003). Selenium and iodine supplementation of rural Tibetan children affected by Kashin-Beck osteoarthropathy. Am. J. Clin. Nutr..

[87] Ning Y.J., Wang X., Ren L., Guo X. (2013). Effects of dietary factors on selenium levels of children to prevent Kashin-Beck disease during a high-prevalence period in an endemic area: A cohort study. Biol. Trace Elem. Res..

[88] Zou K., Liu G., Wu T., Du L. (2009). Selenium for preventing Kashin-Beck osteoarthropathy in children: A meta-analysis. Osteoarthr. Cartil..

[89] Ning Y., Wang X., Wang S., Zhang F., Zhang L., Lei Y., Guo X. (2015). Is It the Appropriate Time to Stop Applying Selenium Enriched Salt in Kashin-Beck Disease Areas in China?. Nutrients.

[90] Institute of Medicine (2000). Dietary Reference Intakes for Vitamin C, Vitamin E, Selenium, and Carotenoids.

[91] Sun L.Y., Meng F.G., Li Q., Zhao Z.J., He C.Z., Wang S.P., Sa R.L., Man W.W., Wang L.H. (2014). Effects of the consumption of rice from non-KBD areas and selenium supplementation on the prevention and treatment of paediatric Kaschin-Beck disease: An epidemiological intervention trial in the Qinghai Province. Osteoarthr. Cartil..

[92] Suetens C., Moreno-Reyes R., Chasseur C., Mathieu F., Begaux F., Haubruge E., Durand M., Nève J., Vanderpas J. (2001). Epidemiological support for a multifactorial aetiology of Kashin-Beck disease in Tibet. Int. Orthop..

[93] Liquiang X., Wangxing S., Qinhua X., Huiming H., Schramel P. (1991). Selenium in Kashin-Beck disease areas. Biol. Trace Elem. Res..

[94] Hartikainen H. (2005). Biogeochemistry of selenium and its impact on food chain quality and human health. J. Trace Elem. Med. Biol..

[95] Aro A., Alfthan G., Varo P. (1995). Effects of supplementation of fertilizers on human selenium status in Finland. Analyst.

